# Discourse-Level Information Recall in Early and Late Bilinguals: Evidence From Single-Language and Cross-Linguistic Tasks

**DOI:** 10.3389/fpsyg.2021.757351

**Published:** 2021-10-26

**Authors:** Isabelle Chou, Jiehui Hu, Edinson Muñoz, Adolfo M. García

**Affiliations:** ^1^School of Foreign Languages, University of Electronic Science and Technology of China, Chengdu, China; ^2^Departamento de Lingüística y Literatura, Facultad de Humanidades, Universidad de Santiago de Chile, Santiago, Chile; ^3^Cognitive Neuroscience Center (CNC), Universidad de San Andrés, Buenos Aires, Argentina; ^4^National Scientific and Technical Research Council (CONICET), Buenos Aires, Argentina; ^5^Global Brain Health Institute, University of California, San Francisco, San Francisco, CA, United States

**Keywords:** bilingualism, age of L2 acquisition, information recall, single-language processing, cross-linguistic processing

## Abstract

Bilingualism research indicates that verbal memory skills are sensitive to age of second language (L2) acquisition (AoA). However, most tasks employ disconnected, decontextualized stimuli, undermining ecological validity. Here, we assessed whether AoA impacts the ability to recall information from naturalistic discourse in single-language and cross-linguistic tasks. Twenty-four early and 25 late Chinese-English bilinguals listened to real-life L2 newscasts and orally reproduced their information in English (Task 1) and Chinese (Task 2). Both groups were compared in terms of recalled information (presence and correctness of idea units) and key control measures (e.g., attentional skills, speech rate). Across both tasks, information completeness was higher for early than late bilinguals. This occurred irrespective of attentional speed, speech rate, and additional relevant factors. Such results bridge the gap between classical memory paradigms and ecological designs in bilingualism research, illuminating how particular language profiles shape information processing in daily communicative scenarios.

## Introduction

Age of second language (L2) acquisition (AoA) has been shown to influence various memory processes in bilinguals (Yoo and Kaushanskaya, 2016; Volkovyskaya et al., 2017; Macmillan et al., 2021). However, most studies have employed random or arbitrary sequences of disconnected stimuli, failing to assess whether AoA impacts a critical aspect of daily communication: the ability to recall information from unfolding discourse. To bridge this gap, we compared the performance of early and late bilinguals (EBs and LBs) on two naturalistic recall tasks with low and high processing demands.

Age of second language (L2) acquisition is the age of first intensive exposure to L2 (Birdsong, 2006; Saito, 2015a). Depending on whether this occurred before or after middle childhood, with cut-offs ranging from 5 to 8years old (Tao et al., 2011; Cardimona et al., 2016; Nair et al., 2016; Bonfieni et al., 2019; Claussenius-Kalman et al., 2020; Sulpizio et al., 2020), persons are respectively classified into EBs and LBs. During non-naturalistic tasks, EBs outperform or differ from LBs in phonological (Archila-Suerte et al., 2012), lexico-semantic (Sabourin et al., 2014; Saito, 2015a,b), and morphosyntactic (Veríssimo et al., 2018) tasks, as well as in executive control (Bonfieni et al., 2019) measures. Given the ubiquitous role of these domains in discourse processing, AoA may also modulate text-level information recall.

Information recall involves evoking recent or distant events and construing them through language (Rubin and Umanath, 2015), as done when we (re)tell a story, a piece of news, or an anecdote. Though typically studied based on lists of disconnected stimulus lists (Raman et al., 2018; Kilecioğlu et al., 2020; Macmillan et al., 2021), this domain can be fruitfully tapped through two naturalistic tasks: single-language recall (SLR) and consecutive interpreting (CI). In SLR tasks, participants are presented with pieces of discourse and asked to recount their contents as exhaustively as possible, in the same language (Hiltunen and Vik, 2017; Prichard and Christman, 2017; Newberry and Bailey, 2019). For its part, CI requires listening to sequences of continuous speech in one language so as to render them into another language after a time period (Hamidi and Pöchhacker, 2007; Choi, 2013; Liu, 2013; Wu, 2013). Both tasks allow for the use of notes to aid information retrieval but they differ in their overarching cognitive demands, with CI proving more stringent than SLR due to the added challenges of cross-linguistic processing (Hiltunen et al., 2016).

Although these two tasks have informed several topics in the bilingualism agenda (Hiltunen and Vik, 2017; Dong et al., 2018; García, 2019; Newberry and Bailey, 2019), no study has examined how AoA impacts on them. Yet, evidence from non-naturalistic studies shows that EBs outperform LBs in cued word (Yoo and Kaushanskaya, 2016; Macmillan et al., 2021) and picture (Volkovyskaya et al., 2017) recall tasks, suggesting that the same may occur in the face of unfolding texts. Moreover, recall of discourse-level information can be influenced by expertise in specific bilingual skills (Hiltunen and Vik, 2017), indicating that this domain can indeed be shaped by subject-level variables in this population.

Importantly, to effectively capture the impact of AoA on information recall, key potential confounds need to be addressed. First, given that AoA may correlate with other bilingual-experience-related variables that impact information recall (e.g., L2 proficiency, time of exposure; Oh et al., 2019; López, 2021), relevant factors should be accounted for in the group-formation stage, ideally through exhaustive, validated instruments. Moreover, AoA is known to modulate speech rate, a factor that could impinge on information delivery upon testing (Guion et al., 2000; Saito, 2015a). Also, AoA can affect attentional skills (Kapa and Colombo, 2013), which may critically influence text-level processing by favoring concentration on and appraisal of both key and secondary information (Meppelink and Bol, 2015; Sauer and Hope, 2016). Therefore, robust testing of our hypothesis should directly tackle these issues.

Against this background, we examined whether information recall is affected by AoA in text-level tasks with low (SLR) and high (CI) cognitive demands. Our analyses focused on a validated completeness measure (exhaustiveness and precision in information recall). To account for key potential confounds, we ensured that both groups were systematically matched across multiple bilingual-experience variables using a validated tool (Schaeffer et al., 2020), while empirically addressing the role of speech rate and attentional speed as potential modulators of participants’ outcomes. Based on previous findings, we hypothesized that, across both tasks, information would be better recalled by EBs than LBs. With this approach, we aim to shed new light on how AoA influences bilinguals’ abilities to recall verbal information from naturalistic discourse beyond isolated stimuli.

## Materials and Methods

### Participants

The study comprised 49s-year students (44 female) from a translating and interpreting program at University of Electronic Science and Technology of China, recruited for course credit. They had received around 64h of consecutive interpreting classes before testing. A power estimation analysis with G*Power 3.1 (Faul et al., 2009) for an ANCOVA (alpha=0.05, power=0.80, *η*_p_^2^=0.25) showed that reliable effects could be obtained with 26 participants. Our actual sample size yielded a power of 0.97. All participants were native Chinese speakers who learned English through formal education or private English tutors. Their ages ranged from 20 to 26years. They were right-handed and none of them had neurological or psychiatric antecedents.

Following a well-established strategy for group formation in bilingualism research (Bartolotti et al., 2011; Vukovic, 2013; Bialystok and Shorbagi, 2021), participants were separated into EBs and LBs based on the median AoA of the whole sample. Crucially, such median value (namely, age 7) is a typical cut-off reported in the literature (Sabourin et al., 2014; Delcenserie and Genesee, 2017; Kousaie et al., 2017; Claussenius-Kalman et al., 2020). Moreover, the resulting groups are well balanced (24 EBs and 25 LBs) and strictly matched for critical sociodemographic, cognitive, and language profile factors (Table 1). Specifically, data acquired before the experimental session through TICQ^1^ (Schaeffer et al., 2020) showed that the two groups differed significantly in their age of L2 acquisition but were matched in terms of age, sex, language competence, interpreting competence, weekly interpreting practice, and key cognitive dimensions – except for attentional speed, which was entered as covariate in all analyses (Table 1). The tasks used in the cognitive assessment protocol are detailed in Supplementary Material.

**Table 1:** Demographic, linguistic, and cognitive profile of the two groups.

	Early bilinguals	Late bilinguals	Early bilinguals vs. late bilinguals	
	*n*=24	*n*=25	value of *p*^a^	Cohen’s *d*
Demographic data				
Sex (F: M)	21:3	23:2	0.63	N/A
Years of age	22.08 (1.28)	22.5 (1.84)	0.36	0.26
Years of education	15.08 (1.28)	15.5 (1.56)	0.37	0.29
Linguistic profile				
Age of L2 learning	6.87 (1.62)	10.58 (1.21)	<0.001	2.58
L2 competence	64.54 (10.12)	64.57 (12.39)	0.96	0.002
Competence in L1-L2 CI	64.07 (12.71)	64.10 (16.52)	0.95	0.002
Competence in L2-L1 CI	63.92 (15.05)	63.94 (12.88)	0.88	0.001
Weekly dedication to L1-L2 CI	4.33 (2.72)	5.64 (7.36)	0.53	0.30
Weekly dedication to L2-L1 CI	3.94 (2.66)	5 (3.36)	0.29	0.34
Competence in L1-L2 translation	64.41 (13.18)	64.08 (18.19)	0.35	0.02
Competence in L2-L1 translation	64.29 (13.80)	64.22 (16.52)	0.75	0.004
Weekly dedication to L1-L2 translation	4.38 (3.08)	5.5 (3.36)	0.34	0.34
Weekly dedication to L2-L1 translation	5.72 (3.84)	5.28 (2.46)	0.34	0.69
Cognitive profile^c^				
Phonemic fluency in L1	16.47 (4.09)	14.87 (3.68)	0.16	0.41
Phonemic fluency in L2	19.41 (6.08)	18.12 (5.02)	0.42	0.23
Semantic fluency in L1	28.25 (8.56)	26.79 (8.63)	0.55	0.16
Semantic fluency in L2	19.25 (5.35)	17.37 (4.17)	0.18	0.39
Digit span	26 (3.61)	25.58 (4.36)	0.72	0.10
Reading span	11.5 (3.40)	10.54 (4.59)	0.41	0.23
Attention: uncued reaction time (ms)^b^	405.80 (47.19)	432.04 (62.22)	0.10	0.47
Attention: uncued error^b^	1.66 (1.40)	1.33 (1.71)	0.46	0.21
Attention: cued reaction time (ms)^b^	356.47 (32.71)	384.29 (50.72)	< 0.05	0.65
Attention: cued error^b^	0.20 (0.50)	0.25 (0.53)	0.78	0.09

Data presented as mean (SD), with gender as an exception. L1, native language (Mandarin Chinese); L2, second language (English); CI, consecutive interpreting; LBs, late bilinguals; and EBs, early bilinguals.

a Based on unpaired two-tailed *t*-tests.

b Based on Posner’s attention task (Posner, 1980).

c Based on validated procedures, not a part of TICQ (Schaeffer et al., 2020).

### Experimental Procedure

Participants performed two tasks in a fixed order: (i) an auditory L2 SLR task, tapping on text comprehension and memory (Bernhardt, 1991; Vander Beken et al., 2020); and (ii) an L2-L1 CI task, capturing interlingual reformulation skills (De Groot and Comijs, 1995; Christoffels et al., 2013). In both cases, they received oral instructions in Chinese (their L1). They sat at desks in a dimly illuminated language lab, with a desktop in front and with no distractions. Stimuli for both tasks were presented binaurally *via* a headset with stereo headphones. Recordings were presented only once. The protocol lasted roughly 20min.

#### SLR Task

The auditory SLR recall task was based on a news report about hygiene efforts during the COVID-19 pandemic in Africa. The report was delivered by a female speaker of American English. The audio clip was downloaded from the Voice of America website^2^ and saved in high-quality mp3 stereo format with 44.1kHz. It lasted 153s, with 352 words produced at a rate of 2.27 per second.

Participants were instructed orally to listen to the recording and then verbally reproduce as much information as they could, in their L2, with all details they could remember. Each participant adjusted the volume to his/her comfort before the test begun. They were allowed to take down any notes they believed necessary with paper and pencil. As in previous studies (Çakmak and Erçetin, 2018; Vander Beken and Brysbaert, 2018; Vander Beken et al., 2020), they were asked to focus on capturing the text’s ideas in their own words rather than provide verbatim renditions. The participants’ speech was recorded through their headset’s built-in microphone and saved as high-quality mp3 files on Xima 3,100 Digital Integrated Language Teaching System V2.0.

#### CI Task

The CI task was based on a news report about a virtual reality spa experience, narrated by a woman in American English. The audio file was also downloaded from the Voice of America website,^3^ and it had the same specifications as the one used in the previous task. The speech lasted 176s and it involved 295 words delivered at a rate of 2.54 per second.

Participants were instructed to listen to the recording and interpret it consecutively into L1. To emulate real-life performance conditions, the recording was stopped twice, with each segment lasting roughly 60s (pauses were made at the same portion of the recording for all participants). Participants were asked to interpret into L1 once the recording stopped, and they were allowed to take down notes with paper and pencil, at will. Their production was recorded exactly as described for the previous task.

### Source-Text Description

In line with reported procedures (Vander Beken and Brysbaert, 2018; Vander Beken et al., 2020), the source texts in each task were first divided into idea units, namely, utterances (typically, phrase-sized constructions) that express a complete idea and contain an actual or tacit verb (Mills et al., 1993; Schiefele and Krapp, 1996; Roediger and Karpicke, 2006; Vander Beken et al., 2020). The number of information units and words, as well as the recordings’ duration and speech rates, are shown in Table 2.

**Table 2:** Idea units per task.

	Task 1 (SLR)	Task 2 (CI)
Number of idea units	37	26
Number of words	351	296
Duration (second)	153	111
Speech rate (word/second)	2.29	2.66

SLR, single-language recall; CI, consecutive interpreting.

### Speech Transcription

Recordings were first automatically transcribed *via* iFlytek,^4^ a software providing 97.5% accuracy in English recognition (Li et al., 2015; Wang et al., 2018). Three individual copy-editors then checked each transcribed file against its recording to ensure optimal quality. The minimal instances requiring editing were acted on by consensus from all three copy-editors. Transcriptions were saved as doc files for further processing and analysis.

### Analysis Measures

#### Information Completeness

This measure captures coarse-grained processes encompassing listening comprehension, higher-order cognitive processing, memory retention, and linguistic reformulation skills (Craik and Lockhart, 1972; Çakmak and Erçetin, 2018; Vander Beken et al., 2020). We first followed standard procedures (Mills et al., 1993) to identify idea units in each naturalistic text. The protocol adopted relies heavily on verbs as the central point in an idea unit. Such an approach captures a key point made in leading linguistic theories (e.g., Systemic Functional Grammar) that frame verbs (and their associated processes) as the key organizing element in the lexico-grammatical and semantic structure of a clause (Halliday, 1994; Halliday and Matthiessen, 2014). We then followed validated scoring protocols to rate the presence and correctness of idea units in each transcription, without focusing on any specific lexical class or construction type (Roediger and Karpicke, 2006; Vander Beken and Brysbaert, 2018; Vander Beken et al., 2020). Each unit was given 1 point if correctly recalled, 0.5 points if partially recalled, and 0 points if incorrectly recalled or omitted.

Such a rating of information completeness does not focus on any particular lexical class. The proportion of correctly recalled information was then calculated over the total number of idea units. As in previous work (Roediger and Karpicke, 2006; Vander Beken and Brysbaert, 2018; Vander Beken et al., 2020), two independent raters scored all recall protocols independently, and a third rater resolved all discrepancies to reach agreement. Inter-rater agreement reached 96% of units for the SLR task and 94% for the CI task. The remaining 4 and 6% of units were resolved in a three-way discussion between both raters and the third rater, following reported procedures (Karpicke and Roediger, 2010; Blunt and Karpicke, 2014).

#### Control Measures

##### Speech Rate

Speech rate is a fluency measure calculated as the number of words spoken in a minute (Hulme et al., 1984; Trofimovich and Baker, 2006; Polyanskaya et al., 2017). The number of words of each transcript was counted in Microsoft Word and the length of each recording was obtained from the corresponding file’s property log. The same procedure was used in both tasks.

##### Additional Measures for the CI Task

We also considered two CI quality measures (Hamidi and Pöchhacker, 2007). In both cases, scores were provided by two independent annotators. The minimal discrepancies that emerged were resolved by a third annotator leading to consensual values. All measures were calculated for each text segment.

First, we measured delivery by quantifying pauses and disfluencies. Following Hamidi and Pöchhacker (2007), we first calculated the mean frequency of filled pause (interjections such as “um,” “uh,” “hmm” or fillers between utterances; Brennan and Williams, 1995), and unfilled pauses (a pause not “filled” by a hesitation form) in each recording. All annotations were marked with Adobe Audition (version 13). Second, in each recording, we calculated the mean frequency of three disfluency metrics: (a) false starts (interruption of a sentence followed by another complete sentence with a change in meaning), (b) repetitions (unwarranted reiteration of a word or a phrase, usually after a pause), and (c) slips of the tongue (deviations from the intended form of an utterance).

Second, we measured quality of expression in terms of grammatical, syntactic, and lexical errors (Hamidi and Pöchhacker, 2007). In line with previous research (Levenston, 1979; Ting et al., 2010), we targeted the following variables: (a) misformation (use of wrong word forms or structures), (b) wrong sentence structure [lack or misuse of a subject and/or a finite (+tense) verb, and/or an independent clause], and (c) wrong word selection (non-native-like word combinations).

### Statistical Analysis

Information completeness and speech rate were compared between groups in each task *via* independent measures ANOVAs. Also, given that EBs and LBs differed in attentional speed (cued reaction time from Posner’s task; see Table 1), the contrasts yielding significant differences were reanalyzed *via* ANCOVAs, including attentional speed outcomes as a covariate, to test whether the effect was driven by the latter factor. As in previous works on SLR and CI (Khateb et al., 2016; Çakmak and Erçetin, 2018; Jost et al., 2018; Vander Beken et al., 2020), for each measure, an outlier detection threshold was set at 3 SDs away from the sample’s mean. No participants were excluded as outliers based on these criteria. Effect sizes were calculated *via* partial eta squared (*η*_p_^2^) for ANCOVAs, with standard benchmarks to discriminate among small (*η*_p_^2^=0.01), medium (*η*_p_^2^=0.06), and large (*η*_p_^2^=0.14) effects (Cohen, 1988). Effect sizes for pairwise comparisons were obtained through Cohen’s *d*. These analyses were run on IBM’s SPSS Statistics (v.26). Also, to further explore the role of attentional differences in our main analyses, we implemented a mediation model per task to examine whether attentional speed mediated the link between AoA and information recall. The mediation analysis provides a quantification of the causal pathways of one or more measurements called mediating variables (Schoemann et al., 2017). Alpha levels were set at *p*<0.05. Mediation analyses were performed on Jamovi (2020), v. 1.2. All experimental data are fully available online (Chou, 2021).

## Results

### SLR Task

**Figure 1:**
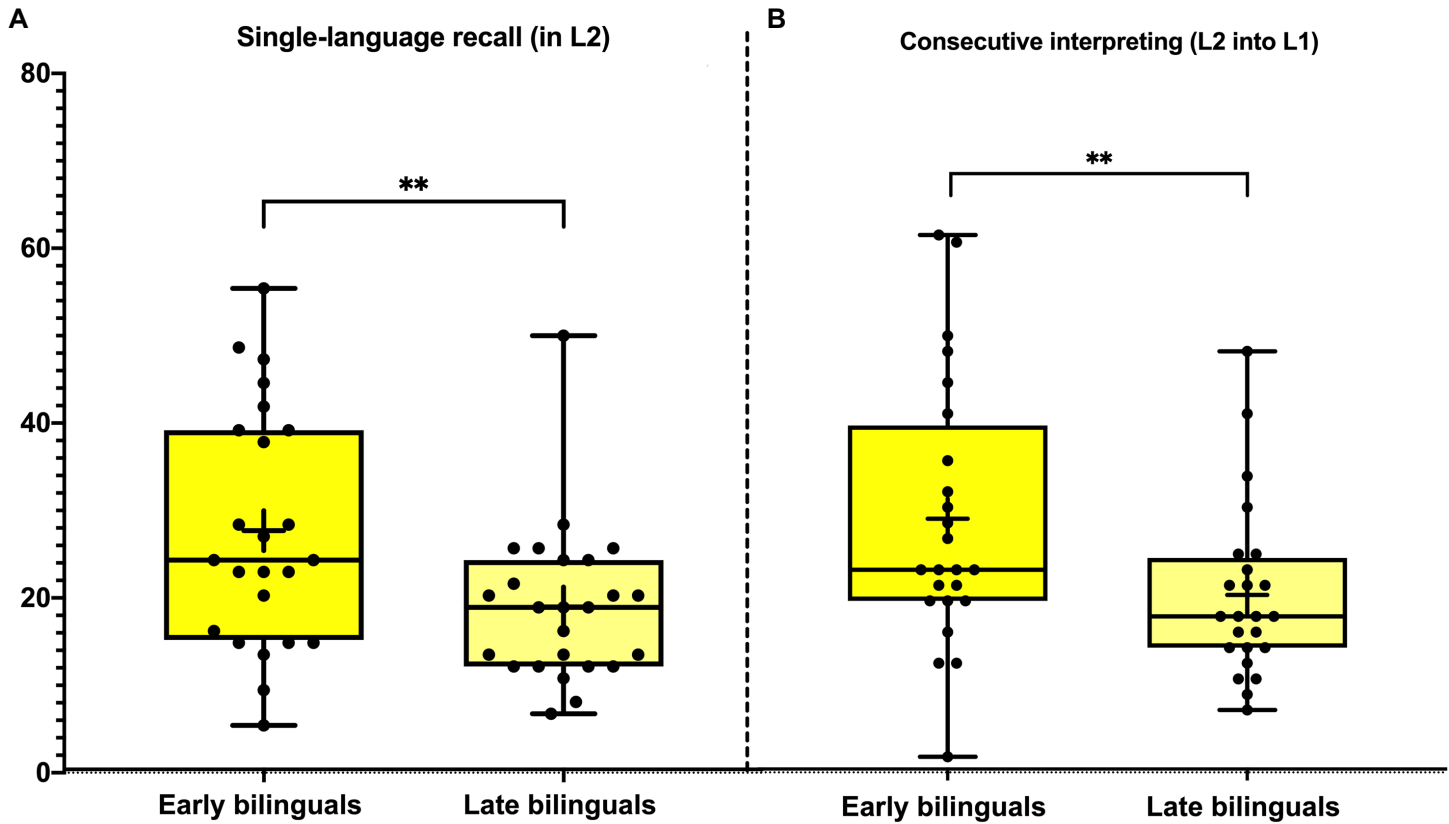
Information recall in naturalistic discourse-level tasks. Early bilinguals correctly recalled more L2 discourse-level information than late bilinguals when tested in L2 **(A)** and in L1 **(B)**. These effects were uninfluenced by attentional speed. The asterisks (**) indicate significant differences at *p*<0.01 for ANCOVA results, covarying for attentional speed.

Concerning information completeness, EBs outperformed LBs [*F*(1, 48)=6.687, *p*=0.01, *η*_p_^2^=0.148], even when covarying for attentional speed [*F*(1, 47)=6.336, *p*=0.01, *η*_p_^2^=0.126; Figure 1A]. Also, a mediation model revealed that the AoA effect on SLR was not mediated by attentional capacity [SE=0.0108, *p*=0.81, *β*=−0.0105, CI (95%)=−0.0237, 0.0187]. The speech rate measure did not differ significantly between groups [*F*(1, 49)=0.27, *p*=0.532, *η*_p_^2^=0.028].

### CI Task

Concerning Information completeness, we found better performance for EBs and LBs [*F*(1, 48)=5.38, *p*=0.02, *η*_p_^2^=0.105], even when covarying for attentional speed [*F*(1, 48)=4.222, *p*<0.05, *η*_p_^2^=0.088; Figure 1B]. A mediation model revealed that the AoA effect on CI was not mediated by attentional capacity [SE=0.0117, *p*=0.59, *β*=−0.0264, CI (95%)=−0.02930, 0.0166]. Speech rate analyses revealed a non-significant effect of group [*F*(1, 47)=1.303, *p*=0.19, *η*_p_^2^=0.016]. Also, none of the five delivery metrics or the quality of expression variables yielded significant differences between groups (for details, see Supplementary Material).

### Correlation Between SLR and CI Outcomes

In an exploratory analysis, we examined whether SLR and CI outcomes were associated in each group. Given that data was normally distributed in both cases, we performed Pearson’s correlations. We observed a significant positive correlation for EBs (*p*<0.001; *r*=0.749) and a non-significant correlation for LBs (*p*=0.586; *r*=0.114).

## Discussion

This study explored the impact of AoA on two naturalistic information recall tasks: SLR and CI. In both cases, information completeness was higher for EBs than LBs, irrespective of attentional speed. No significant differences emerged between groups in any of the control measures. These findings are discussed below.

In SLR, EBs recalled more information than LBs. This aligns with previous studies showing that AoA is associated with information encoding and retrieval in cued word (Yoo and Kaushanskaya, 2016; Macmillan et al., 2021) and picture (Volkovyskaya et al., 2017) recall tasks. Crucially, our study *extends* these findings, suggesting that AoA can also modulate information recall in the face of ecological textual materials. Tentatively, this effect could be influenced by EBs’ greater availability of cognitive resources to meet task-related comprehension and memory demands (Akhtar and Menjivar, 2012). Indeed, lower AoA has been linked to reduced effort for concomitant linguistic (e.g., morphosyntactic; Ullman, 2001) and executive (Akhtar and Menjivar, 2012) processes. This, we propose, might free cognitive resources to meet information retrieval requisites during SLR.

The advantage of EBs was also significant in CI. This task is more demanding than SLR, as information must be first processed in L2, retained for a brief period, and then retrieved and encoded into L1 (Napier, 2015; Liang et al., 2019). Whereas some AoA effects tend to attenuate or disappear as task complexity increases (Marful et al., 2016; Catling and Elsherif, 2020), this result suggests that low AoA boosts information recall even under stringent processing conditions. Of note, better performance for EBs than LBs has been reported in other studies involving tasks with different complexity, such as forward digit recall, passage completion test, and non-word repetition (Delcenserie and Genesee, 2017). In this sense, our study is the first to show that such demand-independent advantages for EBs also manifest for information recall in natural discourse.

Moreover, as shown in Table 1, both groups were systematically matched across numerous variables that could impact on information recall skills, such as language competence (Schweppe et al., 2015) and working memory (Loaiza et al., 2011; Ljung et al., 2013). In particular, such matching was achieved through a recent tool shown to possess high validity to discriminate bilingual individuals based on multiple aspects of their linguistic profile (Schaeffer et al., 2020). In this sense, AoA seems to modulate text-level information recall not only irrespective of task demands, but also regardless of relevant executive and linguistic variables as well as the participants’ broad bilingual and cognitive profiles. This would suggest that AoA has a direct impact on information recall outcomes. Potentially, this might be due to a sum effect of its experiential implications, such as more years of L2 exposure in time windows sensitive to incidental development of linguistic skills (Paradis, 2009). In this sense, our findings pave the way for a new agenda to elucidate the determinants of the observed effect.

Interestingly, EBs also presented enhanced attentional skills. However, ANCOVA results showed that their advantages over LBs in both tasks were uninfluenced by such a factor. Although, attentional capacity may impact information recall in single-item tasks (Sauer and Hope, 2016; Unsworth and Miller, 2021), our findings indicate that, in the face of discourse-level material, the overall effect of AoA on recall may supersede that of attentional skills proper. Indeed, such a finding is in line with evidence that linguistic processing differences between specific bilingual groups are not driven by cognitive control differences (García, 2014; Santilli et al., 2019). This was corroborated by mediation analyses, showing that attentional skills did not mediate the effect of AoA on information recall in either task. Indeed, previous research shows that linguistic processing advantages related to individual bilingual experiences may emerge irrespective of executive outcomes (Archila-Suerte et al., 2015; Santilli et al., 2019). In addition, better information recall for EBs was observed in the absence of speech rate differences, further suggesting that their higher scores were not driven by linguistic productivity.

Further attesting to differences between both groups, SLR and CI outcomes were significantly (and positively) correlated in EBs, but not in LBs. This suggests that individual information recall skills in the former group operate in a task-independent manner, whereas such abilities seem to be differently deployed depending on task difficulty in LBs. Future studies could illuminate this issue with protocols designed specifically to capture cross-task variability in each population.

Of note, participants’ performance was rather low in both conditions, with EBs approaching 30% accuracy. Though somewhat unexpected, this is in keeping with the heterogeneous outcomes reported in the literature, ranging from <45% (Vander Beken and Brysbaert, 2018) to >65% (Hiltunen and Vik, 2017). For example, using L1 and L2 SLR tasks, Vander Beken and Brysbaert (2018) reported scores of 44.1 and 56.3%, respectively. Our samples’ lower performance level probably reflects our protocol’s demands. Whereas Vander Beken and Brysbaert (2018) used written texts and asked participants to summarize them in detail for writing-based recall, the present study involved longer auditory texts with on-the-fly note-taking and ulterior oral recall. Given that oral L2 tasks tend to reduce performance relative to their written counterparts (Kim, 2015; Vandergrift and Baker, 2015).

Finally, our findings carry theoretical implications. First, whereas multiple studies have revealed AoA effects in highly constrained tasks, ours shows that earlier L2 acquisition can impact text-level processing. In this sense, our study meets recent calls for more ecological approaches to the study of language, in general, and bilingualism, in particular (Trevisan et al., 2017; Adams et al., 2018; García et al., 2018; Trevisan and García, 2019; Birba et al., 2020a,b), leading to more situated accounts of the phenomenon. Second, our results reinforce the view that discourse-level recalling is influenced by specific bilingual experiences. Hiltunen and Vik (2017) found that prose recall was better in bilinguals with sustained experience in simultaneous interpreting relative to non-interpreter bilinguals. The present study indicates that AoA may be yet another subject-level variable shaping this domain among the heterogeneous bilingual population. Third, our results show that the impact of AoA *per se* seems strong enough so as to become uninfluenced by other variables known to impact task performance, such as speech rate and attentional skills. This corroborates the view that AoA may represent one of the most important variables accounting for cognitive variability across bilingual individuals (Birdsong, 2018; DeLuca et al., 2020). Briefly, these considerations can inform and extend current accounts of how different bilingual profiles impact cognitive skills.

## Limitations and Avenues for Further Research

Our study is not without limitations. First, our sample size was moderate. Although it was supported by power estimation results and it proved similar to or larger than those of other relevant studies in the field (Sabourin et al., 2014; Giezen et al., 2015; Yoo and Kaushanskaya, 2016), future research should replicate our experiment with more participants. Second, the current study used only two tasks, with brief texts allowing for no interaction. New studies should assess whether AoA impacts recall in longer and interactive pieces of discourse. Third, given our interest on AoA, the present design was focused how bilinguals recall information from L2 texts in both intra- and inter-linguistic conditions. Looking forward, it would be interesting to explore whether and how information recall is affected by AoA during L1 SLR and L1-to-L2 CI. This would provide insights on potential asymmetrical effects known to modulate other aspects of bilingual processing (Xia and Andrews, 2015; Declerck and Grainger, 2017; Olson, 2017). Fourth, our samples contained a majority of female participants. While this captures the gender distribution of the profession in China (where recruitment was conducted; Han, 2016) and beyond (Hickey, 2019), while favoring comparability with earlier research (Injoque-Ricle et al., 2015; Dong and Zhong, 2017; Hiltunen and Vik, 2017; Ünlü and Şimşek, 2018; Santilli et al., 2019), future works should strive to find more balanced samples. Also, our median-split strategy for group formation yielded a cut-off of 7 to separate EBs and LBs. While other thresholds, such as AoA 6 or 7, may prove relevant, these are impracticable in our sample as they give rise to highly unbalanced groups. New studies should explore whether present results are reproduced using other AoA cut-offs. Fifth, participants’ notes were discarded as their analysis was not contemplated in our design. While these data were orthogonal to our hypotheses, future works should systematically collect and analyze these materials as potential mediators of information recall outcomes. Sixth, the use of real-life materials maximized ecological validity but it prevented us from introducing “task” as a within-subject predictor, given that the texts used for SLR and CI differed in many respects. Future works could replicate our study employing validated protocols to create naturalistic texts, which are matched for multiple variables (Trevisan et al., 2017; García et al., 2018; Trevisan and García, 2019; Birba et al., 2020a,b; Moguilner et al., 2021). Seventh, data on AoA were gleaned through self-report assessments. Despite their limitations (e.g., social desirability biases), subjective estimations of bilingual profiles are standard in the field (Ardal et al., 1990; Weber-Fox and Neville, 1996, 2001; Mahendra and Arkin, 2003; Moreno and Kutas, 2005; Hernandez et al., 2007; Pakulak and Neville, 2011; Waldron and Hernandez, 2013; Berken et al., 2015; Nichols and Joanisse, 2016; Santilli et al., 2019; Vilas et al., 2019), and their outcomes can predict language ability (Marian et al., 2007), reproduce reaction-time results (Langdon et al., 2005), and replicate naming test scores (Gollan et al., 2012). Yet, future works should strive to test our hypotheses with objective AoA measures. Finally, low scores were observed across tasks. In addition to task-specific factors (e.g., text length and difficulty, note-talking modality), this might reflect the stringent criteria of our performance judgment protocol. While these criteria have been reported in previous works (Mills et al., 1993; Roediger and Karpicke, 2006; Vander Beken and Brysbaert, 2018; Vander Beken et al., 2020), maximizing comparability between our findings and relevant antecedents, future studies should consider different exigency thresholds.

## Conclusion

Our study suggests that a lower AoA entails better abilities to recall information from naturalistic texts, irrespective of task demands and attentional skills. Such results bridge the gap between classical memory paradigms and ecological designs in bilingualism research. Further work along these lines could afford novel insights on how particular language profiles shape information processing in daily communicative settings.

## Data Availability Statement

All experimental data are fully available online at: https://osf.io/xbszv/.

## Ethics Statement

Ethical review and approval was not required for the study on human participants in accordance with the local legislation and institutional requirements. The patients/participants provided their written informed consent to participate in this study.

## Author Contributions

IC: concept and design, data collection, data analysis, and manuscript draft preparation. JH: concept and design and manuscript revision. EM: manuscript revision. AG: analysis of strategy, manuscript draft preparation, and critical revision. All authors contributed to the article and approved the submitted version.

## Funding

This work was supported by Humanities and Social Science Fund of Chinese Ministry of Education (grant number: 17YJC740023), Foreign Expert Program in China (grant numbers: G20190023033 and G20200023036), CONICET, ANID, and FONDECYT Regular (grant number: 1210176), and Programa Interdisciplinario de Investigación Experimental en Comunicación y Cognición (PIIECC), Facultad de Humanidades, USACH.

## Conflict of Interest

The authors declare that the research was conducted in the absence of any commercial or financial relationships that could be construed as a potential conflict of interest.

## Publisher’s Note

All claims expressed in this article are solely those of the authors and do not necessarily represent those of their affiliated organizations, or those of the publisher, the editors and the reviewers. Any product that may be evaluated in this article, or claim that may be made by its manufacturer, is not guaranteed or endorsed by the publisher.

## Footnotes

^1^Note that, unlike other language competence tools, the TICQ has been statistically validated across different languages. Also, it captures important factors which are absent in other instruments (e.g., weekly dedication to CI and translation in two directions, competence in CI and translation in two directions).

^2^The report can be accessed here: https://www.voanews.com/science-health/coronavirus-outbreak/wash-your-hands-tough-message-across-africa

^3^The report can be accessed here: https://www.voanews.com/episode/virtual-reality-spa-experience-includes-massage-4235156

^4^iFlytek can be accessed here: https://www.iflyrec.com

## References

[ref1] Adams A. M. Glenberg A. M. Restrepo M. A. (2018). Moved by reading in a Spanish-speaking, dual language learner population. Lang. Speech Learn. Serv. Sch. 49, 582–594. doi: 10.1044/2018_LSHSS-16-0032, PMID: 29800066

[ref2] Akhtar N. Menjivar J. A. (2012). Cognitive and linguistic correlates of early exposure to more than one language. Adv. Child Dev. Behav. 42, 41–78. doi: 10.1016/b978-0-12-394388-0.00002-2, PMID: 22675903

[ref3] Archila-Suerte P. Zevin J. Bunta F. Hernandez A. E. (2012). Age of acquisition and proficiency in a second language independently influence the perception of non-native speech. Bilingualism 15, 190–201. doi: 10.1017/S1366728911000125, PMID: 30197550 PMC6124681

[ref4] Archila-Suerte P. Zevin J. Hernandez A. E. (2015). The effect of age of acquisition, socioeducational status, and proficiency on the neural processing of second language speech sounds. Brain Lang. 141, 35–49. doi: 10.1016/j.bandl.2014.11.005, PMID: 25528287 PMC5956909

[ref5] Ardal S. Donald M. W. Meuter R. Muldrew S. Luce M. (1990). Brain responses to semantic incongruity in bilinguals. Brain Lang. 205, 187–205. doi: 10.1016/0093-934x(90)90011-5, PMID: 2224493

[ref6] Bartolotti J. Marian V. Schroeder S. R. Shook A. (2011). Bilingualism and inhibitory control influence statistical learning of novel word forms. Front. Psychol. 2:324. doi: 10.3389/fpsyg.2011.00324, PMID: 22131981 PMC3223905

[ref9] Berken J. A. Gracco V. L. Chen J. K. Watkins K. E. Baum S. Callahan M.. (2015). Neural activation in speech production and reading aloud in native and nonnative languages. NeuroImage 112, 208–217. doi: 10.1016/j.neuroimage.2015.03.016, PMID: 25776210

[ref10] Bernhardt E. B. (1991). Reading Development in a Second Language, Theoretical, Empirical and Classroom Perspective. Norwood, NJ: Ablex.

[ref12] Bialystok E. Shorbagi S. H. (2021). Subtle increments in socioeconomic status and bilingualism jointly affect children’s verbal and nonverbal performance. J. Cogn. Dev. 22, 467–490. doi: 10.1080/15248372.2021.1901711

[ref13] Birba A. Beltrán D. Caro M. M. Trevisan P. Kogan B. Sedeño L.. (2020a). Motor-system dynamics during naturalistic reading of action narratives in first and second language. NeuroImage 216:116820. doi: 10.1016/j.neuroimage.2020.116820, PMID: 32278096 PMC7412856

[ref14] Birba A. Vitale F. Padrón I. Dottori M. de Vega M. Zimerman M.. (2020b). Electrifying discourse: anodal tDCS of the primary motor cortex selectively reduces action appraisal in naturalistic narratives. Cortex 132, 460–472. doi: 10.1016/j.cortex.2020.08.005, PMID: 32950239 PMC7655702

[ref15] Birdsong D. (2006). Age and second language acquisition and processing: a selective overview. Lang. Learn. 56, 9–49. doi: 10.1111/j.1467-9922.2006.00353.x

[ref16] Birdsong D. (2018). Plasticity, variability and age in second language acquisition and bilingualism. Front. Psychol. 9:81. doi: 10.3389/fpsyg.2018.00081, PMID: 29593590 PMC5857581

[ref17] Blunt J. R. Karpicke J. D. (2014). Learning with retrieval-based concept mapping. J. Educ. Psychol. 106, 849–858. doi: 10.1037/a0035934

[ref18] Bonfieni M. Branigan H. P. Pickering M. J. Sorace A. (2019). Language experience modulates bilingual language control: the effect of proficiency, age of acquisition, and exposure on language switching. Acta Psychol. 193, 160–170. doi: 10.1016/j.actpsy.2018.11.004, PMID: 30640064

[ref19] Brennan S. E. Williams M. (1995). The feeling of another’s knowing: prosody and filled pauses as cues to listeners about the metacognitive states of speakers. J. Mem. Lang. 34, 383–398. doi: 10.1006/jmla.1995.1017

[ref20] Çakmak F. Erçetin G. (2018). Effects of gloss type on text recall and incidental vocabulary learning in mobile-assisted L2 listening. ReCALL 30, 24–47. doi: 10.1017/S0958344017000155

[ref21] Cardimona K. Smith P. Roberts L. S. (2016). Lexical organization in second language acquisition: does the critical period matter? TESOL J. 7, 540–565. doi: 10.1002/tesj.219

[ref22] Catling J. C. Elsherif M. M. (2020). The hunt for the age of acquisition effect: it's in the links! Acta Psychol. 209:103138. doi: 10.1016/j.actpsy.2020.103138, PMID: 32805435

[ref23] Choi J. Y. (2013). “Assessing the impact of text length on consecutive interpreting,” in Assessment Issues in Language Translation and Interpreting. eds. Tsagari D. van Deemter R. (Frankfurt am Main: Peter Lang), 85–96.

[ref24] Chou I. (2021). Data from Discourse-level information recall in early and late bilinguals. Open Science Framework (OSF). Available at: https://osf.io/xbszv/ (Accessed September 30, 2021).

[ref25] Christoffels I. K. Ganushchak L. Koester D. (2013). Language conflict in translation: an ERP study of translation production. J. Cogn. Psychol. 25, 646–664. doi: 10.1080/20445911.2013.821127

[ref26] Claussenius-Kalman H. Vaughn K. A. Archila-Suerte P. Hernandez A. E. (2020). Age of acquisition impacts the brain differently depending on neuroanatomical metric. Hum. Brain Mapp. 41, 484–502. doi: 10.1002/hbm.24817, PMID: 31600019 PMC7267963

[ref27] Cohen J. (1988). Statistical Power Analysis for the Behavioral Sciences. New York, NY: Routledge Academic.

[ref28] Craik F. I. M. Lockhart R. S. (1972). Levels of processing: a framework for memory research. J. Verbal Learn. Verbal Behav. 11, 671–684. doi: 10.1016/S0022-5371(72)80001-X

[ref31] De Groot A. M. Comijs H. (1995). Translation recognition and translation production: comparing a new and an old tool in the study of bilingualism. Lang. Learn. 45, 467–509. doi: 10.1111/j.1467-1770.1995.tb00449.x

[ref32] Declerck M. Grainger J. (2017). Inducing asymmetrical switch costs in bilingual language comprehension by language practice. Acta Psychol. 178, 100–106. doi: 10.1016/j.actpsy.2017.06.002, PMID: 28646654

[ref33] Delcenserie A. Genesee F. (2017). The effects of age of acquisition on verbal memory in bilinguals. Int. J. Biling. 21, 600–616. doi: 10.1177/1367006916639158

[ref34] DeLuca V. Rothman J. Bialystok E. Pliatsikas C. (2020). Duration and extent of bilingual experience modulate neurocognitive outcomes. NeuroImage 204, 116–222. doi: 10.1016/j.neuroimage.2019.116222, PMID: 31557543

[ref35] Dong Y. Liu Y. Cai R. (2018). How does consecutive interpreting training influence working memory: a longitudinal study of potential links between the two. Front. Psychol. 9:875. doi: 10.3389/fpsyg.2018.00875, PMID: 29922199 PMC5996275

[ref36] Dong Y. Zhong F. (2017). Interpreting experience enhances early attentional processing, conflict monitoring and interference suppression along the time course of processing. Neuropsychologia 95, 193–203. doi: 10.1016/j.neuropsychologia.2016.12.007, PMID: 27939366

[ref37] Faul F. Erdfelder E. Buchner A. Lang A.-G. (2009). Statistical power analyses using G*power 3.1: tests for correlation and regression analyses. Behav. Res. Methods 41, 1149–1160. doi: 10.3758/BRM.41.4.1149, PMID: 19897823

[ref38] García A. M. (2014). The interpreter advantage hypothesis: preliminary data patterns and empirically motivated questions. Transl. Interpret. Stud. 9, 219–238. doi: 10.1075/tis.9.2.04gar

[ref39] García A. M. (2019). The Neurocognition of Translation and Interpreting. Amsterdam/Philadelphia: John Benjamins.

[ref40] García A. M. Bocanegra Y. Herrera E. Moreno L. Carmona J. Baena A.. (2018). Parkinson’s disease compromises the appraisal of action meanings evoked by naturalistic texts. Cortex 100, 111–126. doi: 10.1016/j.cortex.2017.07.003, PMID: 28764852

[ref41] Giezen M. R. Blumenfeld H. K. Shook A. Marian V. Emmorey K. (2015). Parallel language activation and inhibitory control in bimodal bilinguals. Cognition 141, 9–25. doi: 10.1016/j.cognition.2015.04.009, PMID: 25912892 PMC4466161

[ref42] Gollan T. H. Weissberger G. H. Runnqvist E. Montoya R. I. Cera C. M. (2012). Self ratings of spoken language dominance: a multilingual naming test (MINT) and preliminary norms for young and aging Spanish-english bilinguals. Biling. Lang. Cogn. 15, 594–615. doi: 10.1017/S1366728911000332, PMID: 25364296 PMC4212892

[ref43] Guion S. G. Flege J. E. Liu S. Yeni-Komshian G. (2000). Age of learning effects on the duration of sentences produced in a second language. Appl. Psycholinguist. 21, 205–228. doi: 10.1017/S0142716400002034

[ref44] Halliday M. A. K. (1994). Introduction to Functional Grammar. 2nd *Edn*. London: Edward Arnold.

[ref45] Halliday M. Matthiessen C. (2014). An Introduction to Functional Grammar. London and New York: Routledge.

[ref46] Hamidi M. Pöchhacker F. (2007). Simultaneous consecutive interpreting: a new technique put to the test. Meta 52, 276–289. doi: 10.7202/016070ar

[ref210] Han C. (2016). A survey to profile conference interpreting practice in China. Interpreting 18, 259–272.

[ref47] Hernandez A. E. Hofmann J. Kotz S. A. (2007). Age of acquisition modulates neural activity for both regular and irregular syntactic functions. NeuroImage 36, 912–923. doi: 10.1016/j.neuroimage.2007.02.055, PMID: 17490895 PMC1995424

[ref48] Hickey S. (2019). Women take the mic in conference interpreting. AIIC Blog. Available at: https://aiic.org/document/311/AIICBlog_Jun2019_HICKEY_Women_take_the_mic_in_CI_EN.pdf%20right%20click%20and%20Copy%20Link%20Location (Accessed September 24, 2021).

[ref49] Hiltunen S. Pääkkönen R. Vik G. V. Krause C. M. (2016). On interpreters’ working memory and executive control. Int. J. Biling. 20, 297–314. doi: 10.1177/1367006914554406

[ref50] Hiltunen S. Vik G. V. (2017). Interpreters–experts in careful listening and efficient encoding? Findings of a prose recall test. Int. J. Biling. 21, 194–212. doi: 10.1177/1367006915610657

[ref51] Hulme C. Thomson N. Muir C. Lawrence A. (1984). Speech rate and the development of short-term memory span. J. Exp. Psychol. 38, 241–253. doi: 10.1016/0022-0965(84)90124-3

[ref52] Injoque-Ricle I. Barreyro J. P. Formoso J. Jaichenco V. I. (2015). Expertise, working memory and articulatory suppression effect: their relation with simultaneous interpreting performance. Adv. Cogn. Psychol. 11, 56–63. doi: 10.5709/acp-0171-1, PMID: 26207153 PMC4511188

[ref53] Jamovi. (2020). jamovi. Version 1.2 [software]. Available at https://www.jamovi.org (Accessed September 15, 2021).

[ref54] Jost L. B. Radman N. Buetler K. A. Annoni J. M. (2018). Behavioral and electrophysiological signatures of word translation processes. Neuropsychologia 109, 245–254. doi: 10.1016/j.neuropsychologia.2017.12.034, PMID: 29275005

[ref55] Kapa L. L. Colombo J. (2013). Attentional control in early and later bilingual children. Cogn. Dev. 28, 233–246. doi: 10.1016/j.cogdev.2013.01.011, PMID: 24910499 PMC4044912

[ref56] Karpicke J. D. Roediger H. L. (2010). Is expanding retrieval a superior method for learning text materials? Mem. Cognit. 38, 116–124. doi: 10.3758/MC.38.1.116, PMID: 19966244

[ref57] Khateb A. Pegna A. J. Michel C. M. Mouthon M. Annoni J. M. (2016). Semantic relatedness and first-second language effects in the bilingual brain: a brain mapping study. Bilingualism 19, 311–321. doi: 10.1017/S1366728915000140

[ref58] Kilecioğlu E. Raman I. Raman E. (2020). The influence of age of acquisition on recall and recognition in Alzheimer’s patients and healthy ageing controls in Turkish. Appl. Neuropsychol. Adult 1–10. doi: 10.1080/23279095.2020.1796668 [Epub ahead of print], PMID: 32749164

[ref59] Kim Y. S. (2015). Language and cognitive predictors of text comprehension: evidence from multivariate analysis. Child Dev. 86, 128–144. doi: 10.1111/cdev.12293, PMID: 25174258

[ref60] Kousaie S. Chai X. J. Sander K. M. Klein D. (2017). Simultaneous learning of two languages from birth positively impacts intrinsic functional connectivity and cognitive control. Brain Cogn. 117, 49–56. doi: 10.1016/j.bandc.2017.06.003, PMID: 28648285

[ref61] Langdon H. W. Wiig E. H. Nielsen N. P. (2005). Dual-dimension naming speed and language-dominance ratings by bilingual hispanic adults. Biling. Res. J. 29, 319–336. doi: 10.1080/15235882.2005.10162838

[ref62] Levenston E. A. (1979). Second language acquisition: issues and problems. Interlang. Stud. Bull. 4, 147–160.

[ref63] Li J. Deng L. Haeb-Umbach R. Gong Y. (2015). Robust Automatic Speech Recognition: A Bridge to Practical Applications. Waltham: Elsevier.

[ref64] Liang J. Lv Q. Liu Y. (2019). Quantifying interpreting types: language sequence mirrors cognitive load minimization in interpreting tasks. Front. Psychol. 10:285. doi: 10.3389/fpsyg.2019.00285, PMID: 30833918 PMC6387939

[ref65] Liu M. (2013). “Design and analysis of Taiwan’s interpretation certification examination,” in Assessment Issues in Language Translation and Interpreting. eds. Tsagari D. van Deemter R. (Frankfurt am Main: Peter Lang), 163–178.

[ref66] Ljung R. Israelsson K. Hygge S. (2013). Speech intelligibility and recall of spoken material heard at different signal-to-noise ratios and the role played by working memory capacity. Appl. Cogn. Psychol. 27, 198–203. doi: 10.1002/acp.2896

[ref67] Loaiza V. M. McCabe D. P. Youngblood J. L. Rose N. S. Myerson J. (2011). The influence of levels of processing on recall from working memory and delayed recall tasks. J. Exp. Psychol. Learn. Mem. Cogn. 37, 1258–1263. doi: 10.1037/a0023923, PMID: 21707214 PMC11163964

[ref68] López E. M. D. (2021). A bilingual advantage in memory capacity: assessing the roles of proficiency, number of languages acquired and age of acquisition. Int. J. Biling. 25, 606–621. doi: 10.1177/1367006920965714

[ref69] Macmillan M. B. Neath I. Surprenant A. M. (2021). Re-assessing age of acquisition effects in recognition, free recall, and serial recall. Mem. Cognit. 49, 939–954. doi: 10.3758/s13421-021-01137-6, PMID: 33558995

[ref70] Mahendra N. Arkin S. (2003). Effects of four years of exercise, language, and social interventions on Alzheimer discourse. J. Commun. Disord. 36, 395–422. doi: 10.1016/S0021-9924(03)00048-0, PMID: 12927946

[ref71] Marful A. Gómez-Ariza C. J. Barbón A. Bajo T. (2016). Forgetting “novel” but not “dragon”: the role of age of acquisition on intentional and incidental forgetting. PLoS One 11:e0155110. doi: 10.1371/journal.pone.0155110, PMID: 27163698 PMC4862635

[ref72] Marian V. Blumenfeld H. K. Kaushanskaya M. (2007). The language experience and proficiency questionnaire (LEAP-Q): assessing language profiles in bilinguals and multilinguals. J. Speech Lang. Hear. Res. 50, 940–967. doi: 10.1044/1092-4388(2007/067), PMID: 17675598

[ref73] Meppelink C. S. Bol N. (2015). Exploring the role of health literacy on attention to and recall of text-illustrated health information: an eye-tracking study. Comput. Hum. Behav. 48, 87–93. doi: 10.1016/j.chb.2015.01.027

[ref74] Mills C. B. Diehl V. A. Birkmire D. P. Mou L. C. (1993). Procedural text: predictions of importance ratings and recall by models of reading comprehension. Discourse Process. 16, 279–315. doi: 10.1080/01638539309544841

[ref75] Moguilner S. Birba A. Fino D. Isoardi R. Huetagoyena C. Otoya R.. (2021). Multimodal neurocognitive markers of frontal lobe epilepsy: insights from ecological text processing. NeuroImage 235:117998. doi: 10.1016/j.neuroimage.2021.117998, PMID: 33789131 PMC8272524

[ref76] Moreno E. M. Kutas M. (2005). Processing semantic anomalies in two languages: an electrophysiological exploration in both languages of Spanish—english bilinguals. Cogn. Brain Res. 22, 205–220. doi: 10.1016/j.cogbrainres.2004.08.010, PMID: 15653294

[ref77] Nair V. K. Biedermann B. Nickels L. (2016). Consequences of late bilingualism for novel word learning: evidence from tamil–english bilingual speakers. Int. J. Biling. 20, 473–487. doi: 10.1177/1367006914567005

[ref78] Napier J. (2015). “Comparing signed and spoken language interpreting,” in The Routledge Handbook of Interpreting. eds. Mikkelson H. Jourdinais R. (London: Routledge), 141–155.

[ref79] Newberry K. M. Bailey H. R. (2019). Does semantic knowledge influence event segmentation and recall of text? Mem. Cognit. 47, 1173–1187. doi: 10.3758/s13421-019-00926-4, PMID: 30915653

[ref80] Nichols E. S. Joanisse M. F. (2016). Functional activity and white matter microstructure reveal the independent effects of age of acquisition and proficiency on second-language learning. NeuroImage 143, 15–25. doi: 10.1016/j.neuroimage.2016.08.053, PMID: 27570106

[ref81] Oh T. M. Graham S. Ng P. Yeh I. B. Chan B. P. Edwards A. M. (2019). Age and proficiency in the bilingual brain revisited: activation patterns across different L2-learner types. Front. Commun. 4:39. doi: 10.3389/fcomm.2019.00039

[ref82] Olson D. J. (2017). Bilingual language switching costs in auditory comprehension. Lang. Cogn. Neurosci. 32, 494–513. doi: 10.1080/23273798.2016.1250927

[ref83] Pakulak E. Neville H. J. (2011). Maturational constraints on the recruitment of early processes for syntactic processing. J. Cogn. Neurosci. 23, 2752–2765. doi: 10.1162/jocn.2010.21586, PMID: 20964590 PMC3154972

[ref84] Paradis M. (2009). Declarative and Procedural Determinants of Second Languages. *Vol*. 40. London/New York: John Benjamins Publishing.

[ref85] Polyanskaya L. Ordin M. Busa M. G. (2017). Relative salience of speech rhythm and speech rate on perceived foreign accent in a second language. Lang. Speech 60, 333–355. doi: 10.1177/0023830916648720, PMID: 28915779

[ref86] Posner M. I. (1980). Orienting of attention. Q. J. Exp. Psychol. 32, 3–25. doi: 10.1080/00335558008248231 7367577

[ref87] Prichard E. C. Christman S. D. (2017). Inconsistent-handed advantage in episodic memory extends to paragraph-level materials. Memory 25, 1063–1071. doi: 10.1080/09658211.2016.1257725, PMID: 27868481

[ref88] Raman I. Raman E. İkier S. Kilecioğlu E. Uzun Eroğlu D. Zeyveli Ş. (2018). Differential effects of age of acquisition and frequency on memory: evidence from free recall of pictures and words in Turkish. Writing Syst. Res. 10, 1–14. doi: 10.1080/17586801.2017.1420727

[ref89] Roediger H. L. Karpicke J. D. (2006). Test-enhanced learning: taking memory tests improves long-term retention. Psychol. Sci. 17, 249–255. doi: 10.1111/j.1467-9280.2006.01693.x, PMID: 16507066

[ref90] Rubin D. C. Umanath S. (2015). Event memory: a theory of memory for laboratory, autobiographical, and fictional events. Psychol. Rev. 122, 1–23. doi: 10.1037/a0037907, PMID: 25330330 PMC4295926

[ref91] Sabourin L. Brien C. Burkholder M. (2014). The effect of age of L2 acquisition on the organization of the bilingual lexicon: evidence from masked priming. Bilingual. Lang. Cogn. 17:542. doi: 10.1017/S1366728913000643

[ref92] Saito K. (2015a). The role of age of acquisition in late second language oral proficiency attainment. Stud. Sec. Lang. Acquis. 37, 713–743. doi: 10.1017/S0272263115000248

[ref93] Saito K. (2015b). Experience effects on the development of late second language learners’ oral proficiency. Lang. Learn. 65, 563–595. doi: 10.1111/lang.12120

[ref94] Santilli M. Vilas M. G. Mikulan E. Caro M. M. Muñoz E. Sedeño L.. (2019). Bilingual memory, to the extreme: lexical processing in simultaneous interpreters. Bilingualism 22, 331–348. doi: 10.1017/S1366728918000378

[ref95] Sauer J. Hope L. (2016). The effects of divided attention at study and reporting procedure on regulation and monitoring for episodic recall. Acta Psychol. 169, 143–156. doi: 10.1016/j.actpsy.2016.05.015, PMID: 27311110

[ref96] Schaeffer M. Huepe D. Hansen-Schirra S. Hofmann S. Muñoz E. Kogan B.. (2020). The translation and interpreting competence questionnaire: an online tool for research on translators and interpreters. Perspectives 28, 90–108. doi: 10.1080/0907676X.2019.1629468

[ref97] Schiefele U. Krapp A. (1996). Topic interest and free recall of expository text. Learn. Individ. Differ. 8, 141–160. doi: 10.1016/S1041-6080(96)90030-8

[ref98] Schoemann A. M. Boulton A. J. Short S. D. (2017). Determining power and sample size for simple and complex mediation models. Soc. Psychol. Pers. Sci. 8, 379–386. doi: 10.1177/1948550617715068

[ref99] Schweppe J. Barth S. Ketzer-Nöltge A. Rummer R. (2015). Does verbatim sentence recall underestimate the language competence of near-native speakers? Front. Psychol. 6:63. doi: 10.3389/fpsyg.2015.00063, PMID: 25698996 PMC4316704

[ref100] Sulpizio S. Del Maschio N. Del Mauro G. Fedeli D. Abutalebi J. (2020). Bilingualism as a gradient measure modulates functional connectivity of language and control networks. NeuroImage 205:116306. doi: 10.1016/j.neuroimage.2019.116306, PMID: 31654763

[ref101] Tao L. Marzecová A. Taft M. Asanowicz D. Wodniecka Z. (2011). The efficiency of attentional networks in early and late bilinguals: the role of age of acquisition. Front. Psychol. 2:123. doi: 10.3389/fpsyg.2011.00123, PMID: 21713011 PMC3114252

[ref102] Ting S. H. Mahadhir M. Chang S. L. (2010). Grammatical errors in spoken english of university students in oral communication course. J. Lang. Stud. 10, 53–69.

[ref103] Trevisan P. García A. M. (2019). Systemic functional grammar as a tool for experimental stimulus design: new appliable horizons in psycholinguistics and neurolinguistics. Lang. Sci. 75, 35–46. doi: 10.1016/j.langsci.2019.101237

[ref104] Trevisan P. Sedeño L. Birba A. Ibáñez A. García A. M. (2017). A moving story: whole-body motor training selectively improves the appraisal of action meanings in naturalistic narratives. Sci. Rep. 7:12538. doi: 10.1038/s41598-017-12928-w, PMID: 28970538 PMC5624907

[ref105] Trofimovich P. Baker W. (2006). Learning second language suprasegmentals: effect of L2 experience on prosody and fluency characteristics of L2 speech. Stud. Sec. Lang. Acquis. 28, 1–30. doi: 10.1017/S0272263106060013

[ref106] Ullman M. T. (2001). The neural basis of lexicon and grammar in first and second language: the declarative/procedural model. Bilingual. Lang. Cogn. 4, 105–122. doi: 10.1017/S1366728901000220

[ref107] Ünlü E. A. Şimşek Ç. S. (2018). Testing the impact of formal interpreting training on working memory capacity: evidence from Turkish–english students–interpreters. Lingua 209, 78–88. doi: 10.1016/j.lingua.2018.04.003

[ref108] Unsworth N. Miller A. L. (2021). Encoding dynamics in free recall: examining attention allocation with pupillometry. Mem. Cognit. 49, 90–111. doi: 10.3758/s13421-020-01077-7, PMID: 32761311

[ref7] Vander Beken H. Brysbaert M. (2018). Studying texts in a second language: the importance of test type. Bilingual. Lang. Cogn. 21, 1062–1074. doi: 10.1017/S1366728917000189

[ref8] Vander Beken H. De Bruyne E. Brysbaert M. (2020). Studying texts in a non-native language: a further investigation of factors involved in the L2 recall cost. Q. J. Exp. Psychol. 73, 891–907. doi: 10.1177/1747021820910694, PMID: 32065046

[ref109] Vandergrift L. Baker S. (2015). Learner variables in second language listening comprehension: an exploratory path analysis. Lang. Learn. 65, 390–416. doi: 10.1111/lang.12105

[ref110] Veríssimo J. Heyer V. Jacob G. Clahsen H. (2018). Selective effects of age of acquisition on morphological priming: evidence for a sensitive period. Lang. Acquis. 25, 315–326. doi: 10.1080/10489223.2017.1346104

[ref111] Vilas M. G. Santilli M. Mikulan E. Adolfi F. Caro M. M. Manes F.. (2019). Reading Shakespearean tropes in a foreign tongue: age of L2 acquisition modulates neural responses to functional shifts. Neuropsychologia 124, 79–86. doi: 10.1016/j.neuropsychologia.2019.01.007, PMID: 30664853

[ref112] Volkovyskaya E. Raman I. Baluch B. (2017). Age of acquisition (AoA) effect in monolingual Russian and bilingual Russian (L1)-English (L2) speakers in a free recall task. Writing Syst. Res. 9, 148–163. doi: 10.1080/17586801.2017.1405136

[ref113] Vukovic N. (2013). “When words get physical: evidence for proficiency-modulated somatotopic motor interference during second language comprehension.” in *Proceedings of the Annual Meeting of the Cognitive Science Society*. *Vol. 35*; July 31–August 3, 2013.

[ref114] Waldron E. J. Hernandez A. E. (2013). The role of age of acquisition on past tense generation in Spanish–english bilinguals: an fMRI study. Brain Lang. 125, 28–37. doi: 10.1016/j.bandl.2013.01.002, PMID: 23454071 PMC3624750

[ref115] Wang H. Pan J. Cong L. (2018). Research development and forecast of automatic speech recognition technologies. Telecommun. Sci. 34, 1–11. doi: 10.11959/j.issn.1000-0801.2018095

[ref116] Weber-Fox C. M. Neville H. J. (1996). Maturational constraints on functional specializations for language processing: ERP and behavioral evidence in bilingual speakers. J. Cogn. Neurosci. 8, 231–256. doi: 10.1162/jocn.1996.8.3.231, PMID: 23968150

[ref117] Weber-Fox C. M. Neville H. J. (2001). Sensitive periods differentiate processing of open and closed-class words. J. Speech Lang. Hear. Res. 44:1338. doi: 10.1044/1092-4388(2001/104), PMID: 11776369

[ref119] Wu F. S. (2013). “How do we assess students in the interpreting examinations?” in Assessment Issues in Language Translation and Interpreting. eds. Tsagari D. van Deemter R. (Frankfurt am Main: Peter Lang), 15–33.

[ref200] Xia V. Andrews S. (2015). Masked translation priming asymmetry in Chinese–English bilinguals: making sense of the sense model. Q. J. Exp. Psychol. 68, 294–325. doi: 10.1080/17470218.2014.944195 25014131

[ref120] Yoo J. Kaushanskaya M. (2016). Serial-position effects on a free-recall task in bilinguals. Memory 24, 409–422. doi: 10.1080/09658211.2015.1013557, PMID: 25730660 PMC4633394

