# Supplementary material for: Discourse-Level Information Recall in Early and Late Bilinguals: Evidence From Single-Language and Cross-Linguistic Tasks
Source: Front Psychol. 2021 Oct 26;12:757351. doi: 10.3389/fpsyg.2021.757351 (PMC8576450; doi:10.3389/fpsyg.2021.757351)
Supplement: Supplementary file 1 [file Table_1.DOCX]

Supplementary Material

# Results from the control measures for the CI task

**Table S1.** Descriptive statistics for all measures in the CI task.

| **Measure** | **Early bilinguals** | **Late bilinguals** | **Statistical results** |
| --- | --- | --- | --- |
| **Delivery** |  | |  |
| Unfilled  Pauses | 2.50 (2.91) | 2.16 (1.85) | *F*(1, 47) = 45.964, *p* = .99, η_p_^2^ = .505 |
| Filled  Pauses | 10.41 (3.98) | 14.04 (14.06) | *F*(1, 47) = 0.504,  *p* = .48, η_p_^2^ = .011 |
| False  Starts | 2 (1.77) | 1.29 (1.30) | *F* (1, 48) = 2.496, *p* = .12, η_p_^2^= .051 |
| Repetition | 3.25 (3.39) | 4.17 (4.08) | *F*(1, 48) = 0.716,  *p* = .40, η_p_^2^ = .015 |
| Slips  of the tongue | 1.04 (1.46) | 1.00 (1.10) | *F*(1, 47) = 0.312,  *p* = .579, η_p_^2^ = .007 |
| **Quality of expression** | | |  |
| Misformation | 5.88 (3.43) | 7.42 (4.29) | *F*(1, 47) = 1.742,  *p* = .180, η_p_^2^ = .074 |
| Wrong  sentence order | 0.08 (0.41) | 0.08 (0.28) | *F*(1, 47) = 1.145,  *p* = .240, η_p_^2^ = .030 |
| Lack/misuse  of independent clause | 1.00 (1.21) | 1.21 (1.50) | *F*(1, 47) = 1.180,  *p* = .285, η_p_^2^ = .026 |
